# National Board of Health

**Published:** 1879-05

**Authors:** 


### NATIONAL BOARD OF HEALTH.

---

The Board convened in Atlanta, May 5, at 10. A. M.

The following is a full roll of the members:

President, Dr. J. L. Cabell, of the University of Virginia; Vice-president, Dr. J. S. Billings, Surgeon of the United States army; Secretary, Dr. T. J. Turner, of the United States navy.

Members—Dr. S. Smith, of New York; Dr. H. J. Bowditch, of Boston; Dr. R. W. Mitchell, of Memphis; Dr. S. M. Bemiss, of New Orleans; Dr. T. S. Verdi, of Washington; Dr. P. H. Bailhache, of the United States Marine Hospital; Dr. H. A. Johnson, of Chicago. Hon. S. L. Phillips, of the attorney-general's office, is the legal adviser of the board. There was a full attendance yesterday except Dr. Bowditch and Mr. Phillips. Both are necessarily absent. There were also present a number of leading physicians and sanitarians from all parts of the country, who came by invitation of the board.

Dr. Cabell called the meeting to order and said:

"In behalf of the national board of health, I desire to say that in asking a conference with leading sanitarians from various States, we were animated by a sincere desire to have the benefit of the counsel and advice, as well as the sympathy, of all whose studies have led them to the investigation of sanitary questions. The constituting act looks to such a cause, for after declaring that the board shall consist of seven members from civil life and four from the departments of the United States, it goes on to contemplate and direct a general conference, with students of sanitary laws and observers of their action in various parts of the country." The President here read an exposition as to the studies and methods of the board. He then mapped out the course of a general consultation with sanitarians from all parts of the Union by which the board hoped to be more efficacious.

Dr. Billings was asked to read the programme which had been arranged by the committee appointed to prepare a schedule for the present meeting.

He announced that the meeting of the day would be restricted to the subject of a maritime quarantine and would not touch on those other subjects which would be reserved for other sessions and separate schedules. The Chair called on Dr. Vanderpoel, the inspector of the port of New York. In response, Dr. Vanderpoel addressed the meeting. He said he did not at first think a national quarantine practicable. He had changed his views on the subject. He thought a national quarantine could be well established on the broad principle that there should be general sanitary laws for all the ports, the police regulations of each port to be left to itself. This he deemed the only solution of the delicate question of the relations of the States to general powers of the Union. He announced the proposition that quarantine does not recognize detention as a necessity, except so far as was needed for a thorough examination of the crew, passengers, and cargo, and their purification if need be. He regretted that there was such a prejudice against quarantine, and that it was regarded as a synonym of detention.

In discussing the question, Dr. Vanderpoel said he had known vessels to go to pestilential ports and come back perfectly pure, while others came away full of disease. The great secret was in the manner in which captains attended to their ships. Perfect cleanliness and close watch of habits of the men, would obviate nearly all danger from pestilential ports. Yellow fever always seeks the bilge water, and if this be constantly kept pure, there is little danger. The compulsory cleanliness of all the men would be an additional guarantee of safety.

Dr. Cleeman, a representative of the port of Philadelphia, said foreign vessels were closely watched at that port, and the limit of quarantine was twenty days.

Dr. Billings asked if the question could not be presented to owners of ships so that it would be a matter of self-interest to insure cleanliness.

Dr. Vanderpoel said this could harldly be done in New York, as vessels in quarantine had little inconvenience or loss.

Dr. Howard, of Baltimore, spoke for that port. He said the Brazilian vessels there carried out all necessary precautions, and were very neat. The West Indian vessels were filthy, but he, as inspector, saw that they were cleaned thoroughly. He said there was a strong commercial rivalry between some great cities, and this reason often caused vessels to sail after a slovenly cleansing, so as to get out as soon as vessels from other ports. He doubted if germs of disease seek the bilge. He found that they rather sought dirty clothing.

Dr. Nash, of Norfolk, agreed with Dr. Vanderpoel entirely in his views.

Dr. Cobb, of Pensacola, took a view practically the same as that of Dr. Vanderpoel, as to source of disease and the efficacy of cleanliness. He gave some very interesting experience on the development of fever in a vessel.

Dr. Bemiss, of New Orleans, said that Drs. White and Chapin would come to-day from New Orleans, and could speak fully for that port.

Dr. Billings explained why he had asked the queston of commercial advantage as a motive for cleanliness.

Dr. Vanderpoel said the question must be one of sanitary and not commercial importance. He then advanced the second point in the schedule, namely:

The vessel after it has arrived in a domestic port, from an infected port, and its sanitary treatment.

He gave the method he adopted in New York, showing that it required cleanliness of the ship which was detained forty-eight hours and subjected to fumigation, through airing and other methods of purification, such as perfect scouring.

Dr. Cleeman gave the method at the port of Philadelphia.

Dr. Vanderpoel said that there never was a space of three days in the summer when there was not yellow fever in the port of New York, and yet it was kept out of the city by a thorough quarantine.

Dr. Howard, of Baltimore, gave the system in use at Baltimore which differed from that at New York. He did not see much use in lightening or scouring out the hull of a vessel.

Dr. Billings asked if there were any specific rule for fumigation by gas and the quantity to be employed. Dr. Vanderpoel said it depended on the size of the vessel. Drs. Nash and Howard gave their methods also.

Dr. Turner, of the Board, also spoke as to the proper method of fumigation.

Dr. Dowell, of Galveston, gave the sanitary methods employed in that port.

Dr. Blaine, of Brunswick, Georgia, said he agreed with the general course of the statements made. He said the Southern ports could not depend on a quarantine during the summer months alone. He gave some interesting statements as to the good effects of neatness. He favored a quarantine all the year around for Southern ports. Said he used the "Charleston disinfectant," a mixture which was found to act well.

Dr. Cobb gave the method of inspection and cleanliness at Pensacola, where nine vessels out of ten came with ballast, as it was a port of export nearly altogether. The chief danger was from the discharge of ballast with vegetable matter in it. He stated some of the difficulties in maintaining a perfect quarantine with the limited means of the city.

Dr. Howard spoke of some of the difficulties in keeping a perfect quarantine in Baltimore.

Dr. Campbell, of Augusta, said he agreed that the quarantine ought to be continued all the year in Southern ports, or at least much longer than it is needed in Northern ports.

Dr. Billings moved that the Board adjourn to meet at 3 o'clock in the Senate Chamber.

The Board so adjourned.

AFTERNOON SESSION.

The afternoon session was called to order in the Senate Chamber by President Cabell.

The President said that the discussion of the morning had related only to sailing vessels in port, and the method of their quarantine. The discussion of the afternoon would relate to the treatment of steamers in port.

The discussion was opened by Dr. Vanderpoel, of New York. He said there was much less danger in steamers than in sailing vessels. There are many reasons for this, such as swiftness in sailing, making short ocean trips, lying in infected ports a short time, better ventilation, better order in all departments. He gave the method of treating quarantine steamers in New York, which was very thorough, and bore testimony to its efficacy in preventing all spread of epidemics.

Dr Howard, of Baltimore, agreed with Dr. Vanderpoel as to the relatively greater safety of steamers as compared with sailing vessels. Said few steamers came with cargo to Baltimore.

Dr. Folsom, of Boston, agreed with those views, and so did Dr. Nash, of Norfolk.

Dr. Cleeman, of Philadelphia, said fever seldom came from steamers, and they were treated in Philadelphia about the same as in New York.

Dr. Blaine, of Brunswick, said he had little experience with infected steamships.

Dr. Cobb, of Pensacola, spoke of the method for the quarantine of steamers in Florida.

Dr. White, of New Orleans, spoke of the precautions used in that city for infected steamers. He urged quarantine "in transition" as the only means of perfect safety in Southern ports. He believed the local authorities should be clothed with plenty of power.

Dr. Dowell, of Galveston, spoke for that port, giving the method employed there with usual success. One ship from Rio last year was detained the whole summer.

Dr. Pope, of Fernandina, sustained the statement of Dr. Vanderpoel about steamers from that city to New York, but said he did not give them bills of health.

Dr. Howard, of Baltimore, spoke of the uncertain evidence of bills of health as guarantees of safely.

The President announced that Dr. Chancellor, of Baltimore, had offered a paper on "The Plague," with special reference to the quarantine. On motion, Dr. Chancellor was requested to read his paper and he complied. He said the assembly was not to consider one epidemic disease. Last year the yellow fever carried death into the households of the Mississippi valley. The epidemic had attacked alternately the Atlantic coast and then the Mississippi valley. He said there are other dangers than yellow fever. There are cholera and the plague to be dreaded. He had investigated the question of the plague, and wished to bring to notice a danger which might press itself on us within six months. He had different ideas of quarantine from those generally received. The great need is not to stop commerce, but to enforce strict sanitary laws in cities. His paper was an inquiry into the history of the plague, but referred especially to the proper methods of quarantine against it. His paper was very able and interesting. It was twenty-five minutes long, and held the close attention of the board and spectators. The position taken in the paper was that no epidemics like yellow fever and the plague are contagious from the person or the clothing. They can only spread when the condition of the air is favorable, and therefore there is no reason for a quarantine. The source is back of this. It is in the condition of the cities where the epidemic is to spread. He said the support of a great national quarantine would be stupendous folly if it tried to keep out epidemics by excluding merchandise.

Dr. Vanderpoel said the time allowed the gentleman was out, and he called him to order.

The chair said the question was with the house.

The house gave him permission, and he proceeded to conclude his paper.

Dr. Pope, of Fernandina, made some interesting statements as to the origin of the fever in that place. He believed the fever originated in Fernandina, and that no case had been found on any vessel arriving. He believed that the poison was carried by the atmosphere and that it was generated in the town.

Dr. Howard, of Baltimore, made some explanations as to the quarantine regulations of that city.

The Board then adjourned to 10 o'clock to-morrow.

---

SECOND DAY'S SESSION—TUESDAY, MAY 6TH.

The Board was called to order in the Senate Chamber at 10 o'clock, A.M., by President Cabell. He announced that as the discussion upon maratime quarantine had been completed on the day preceding, the subject before the Board to-day would be inland quarantine. Of the various topics under this head that were discussed yesterday, was

(1.) River transportation.

(a.) How to secure a steamer freedom from infection at New Orleans.

(c.) How to deal with steamers at ports along the Mississippi and its tributaries.

An expression of opinion from Dr. White, of New Orleans, was called for. Dr. White stated that the only way he saw of stopping the infection of a vessel in the port of New Orleans was to prevent it from coming alongside an infected vessel. The law of Louisiana provides that an infected ship shall be removed from port to the quarantine station; so that ships rarely become infected from contact with infected ships in port. A ship that had been infected should be thoroughly cleaned up and the hold whitewashed before she was allowed to go up the Mississippi.

At this point Governor Colquitt arrived, was introduced by President Cabell, and sat through the remainder of the session.

Mr. Eicklehart, of Vicksburg, agreed with Dr. White's views, but thought that an infected vessel should be sunk just below the hull for disinfection.

Dr. Chaille, of New Orleans, remarked that if they accepted the general belief that textile fabrics are a source of great danger in transmitting the fever, the great question was not what should be done with the hull of a vessel, but after that what measures should be taken that these materials shall not be transmitted to ports above. Called for speeches on the question.

Dr. Mitchell, of Tennessee, said that the only resource Tennessee had against the fever was to establish a total non-intercourse with the infected ports; 99-100ths of the people believe in quarantine. It was humilitating to admit that this was the only preventive. In 1873 Memphis was infected from a barge, and doubtless in the same manner last year. River commerce needs protection from this source of infection. Dr. Mitchell also advocated the feasibility of a system of stations of inspection to be located along the Mississippi—so that it could be ascertained if a vessel become infected between ports. He was satisfied that total non-intercourse provided protection against infection, but that preventive was undesirable; people become affected with the fever thus carried where the air is in proper condition for its propagation.

Dr. J. G. Westmoreland desired to ask Dr. Mitchell what particular condition of the atmosphere is necessary to the propagation of yellow fever. Ruled out of order.

Mr. Nash, of Norfolk, stated that during the epidemic in Richmond and Lynchburg, Norfolk kept up continual intercourses with these cities by transferring freight at Hampton Roads. Thought the same system might be practicable on the Mississippi river.

Dr. Rauch, of Illinois, made quite a lengthy speech in support of the theory that the treatment of inland commerce should be analagous to that of foreign commerce. The prevention of the introduction of infectious diseases should be the work. Under ordinary circumstances a steamer should be examined at New Orleans before being allowed to go up the river. In times of an epidemic extra precautions should be taken. Stations should be located along the Mississippi for the care of the sick and for the disinfection of vessels. It is barbarous the way passengers and crews are treated in the time of an epidemic. If the captain cannot land them in cities, they land them in swamps or anywhere along the river. Humanity demands a system of inland prevention as well as a foreign quarantine.

Dr. Pinckney Thompson, of Kentucky, stated that to his mind it was clear that yellow fever can be carried from place to place in textile fabrics. What can then be done by up-river towns when fever is in New Orleans but to prevent steamers from landing? There are but two resources for these towns. One, to keep themselves in such a sanitary condition as to render the introduction of yellow fever harmless; the other to insist that New Orleans keeps the yellow fever from her ports just as New York does. When fever is not in New Orleans, the towns along the river are always safe.

Dr. A. N. Bell, of New York, who had been repeatedly called for during the morning, now arose and cited the experiments that he had made in heat disinfection. He stated that thirty-one years ago he was called upon to take measures against the yellow fever on a schooner that came from a point where the disease had been raging two years. For three hours he steamed the ship under as intense heat as the boilers would give; then he scraped and painted the vessel, and three weeks later no case of sickness was on board. A year later the ship went on a cruise in the very worst fever ports of the West Indies and no case of fever was developed on board. At the same time and place he treated a gunboat in the same manner with the same results. Some time later two boats were taken from the same squadron to New York, passed quarantine, but, though no cases of fever had been aboard for months, the men engaged in remodeling them in a New York dock, were infected with the fever. These boats had not been disinfected by the steam process. Other experiments in steam disinfectants which Dr. Bell has since made in person were cited, and though there have been some failures, he attributes them to a lack of thoroughness of process. Steam heat is preferable to dry heat because the former can be used with less damage to goods.

Dr. Jones then moved that the Board adjourn in order to hear the address of the President of the Medical Association, and that they meet to-morrow morning at 9 o'clock.

---

THIRD DAY'S SESSION—WEDNESDAY, MAY 7.

The National Board of Health convened early this morning, President Cabell's gavel sounding at 9½ o'clock. There were present in the Senate chamber, besides the members of the Board, the Sanitary Council of the Mississippi valley and representatives of many of the State Boards of Health that had been invited to this conference. Before the order of business was taken up, Dr. Rauch, Secretary of the Sanitary Committee of the Mississippi valley, requested to be permitted to report to the Board a series of propositions concerning river transportation, which had been adopted by the council on the preceding evening.

Dr. Vanderpoel opened the debate. He said that the difficulties to be overcome by inland quarantines were much greater than those to be surmounted by foreign quarantine, etc.

Dr. Pickney Thompson suggested that before the Board of Health adopts these propositions they should settle at what point up the river the inspection of a vessel should be stopped. Inspection stations should be well up the river.

Dr. Vanderpoel said that even when a vessel from an infected port showed a clean bill of health all the way up the river, she should at her destination be obliged to unload aside from the wharf, and have her hold thoroughly cleansed.

Dr. White made a few suggestions in regard to bills of health. He said that when a report reaches an up river town that there is fever in New Orleans the impression immediately became universal in said town that New Orleans was a nest of disease; while the facts of the case are that fever usually breaks out in one spot and people within one square of it are safe. The levee may not become infected for some time. Now, though the fever were in New Orleans, if the captain could state in his bill of health that no fever had been within a mile of his vessel she would be considered safe.

Dr. Jones asked if cargoes should not be disinfected before they were delivered to the consignees. He thought it would be well to specify in these propositions what disposition should be made of cargoes at their destination.

Dr. A. N. Bell now seized an opportunity to say a word against some suggestions that had been casually made in one of the speeches of the debate about whitewashing as a preventative of yellow fever. If lime must be used, use it unslaked. In measures against yellow fever, dead lime should be done away with, as well as all other dead matter.

Dr. Plunkett, President of the Sanitary Council of the Mississippi valley, said the idea was general in the Mississippi valley that the "Plymouth" had been frozen out, and that cold would exterminate germs of the yellow fever. It would pacify the public mind if the question of cold as a disinfectant was setteled.

Dr. Turner replied that the inspection of the Plymouth was made by breaking up her decks, cleansing and freezing her, but temperature was not noted by medical officers; the lowest recorded temperature was 7° centigrade. Yet a case originated on her in March last on the starboard side of the vessel, and quite recently, a case has occurred in a corresponding quarter of vessel on the opposite side. It is probable that the water was frozen in the bilges and thawed out by steam.

Dr Rauch suggested that if the medical officer of a vessel was responsible for her condition, instead of the captain, the fever would be prevented. Thought it was important that the Board should take some action towards instituting the change.

Dr. Guion, of the United States Navy, said Dr. Rauch was right. The medical officer of a vessel could only make suggestions. The captain is responsible for the sanitary condition of the ship, and even puts men on the sick list.

Dr. Plunkett here insisted on an answer to his question, whether or not the Plymouth had been frozen out?

Dr. Guion replied that she probably had, though he could not answer until an official report has been made.

Dr. Blaine closed the debate upon the propositions by remarking that it was not safe to depend upon the condition of passengers and crew at the time a vessel arrives at her destination.

The next subject of discussion was now announced by President Cabell.

II. Railroads—(a) Sanitary condition of depots and stations. (b) Quarantine stations on railroads, their positions and organization; specify for passenger trains, sleeping cars, car construction, freight trains, construction trains. (c) Mails. (d) Expressage.

Dr. Kedzie offered on this subject the following resolution:

The depots and stations of railroads and their surroundings shall be kept in a sanitary condition, free from all stagnant water and decomposing organic matter; the local railroad agent or official shall daily inspect the water closets, and shall cause the floor, seats and urinals of such water closets to be emptied at least every three months, and from the first of May to the first of November, he shall cause the privy vaults to be disinfected every week by pouring into every vault a solution of twenty to fifty pounds of sulphate of iron or sulphate of zinc, or ten to fifteen pounds of chloride of lime.

Dr. Kedzie said that fever often germinated in the North from causes that the resolution does away with. Germs of disease from decomposition are carried into the air and then through the air to quite a distance. Cholera propagated in the same way.

Dr. Howard spoke of the importance of railway travel in the yellow fever question.

Dr. Kedzie here offered a resolution entitled "ventilation on cars," as follows:

"All railroad cars should at all times be well ventilated; the freight cars, when loaded, should have bored doors to permit the free entrance of air at all times, whether moving on the track or placed upon the sidings; the passenger and sleeping cars should be provided with automatic ventilators, so as to secure a rapid change of air in the cars at all times.

"The upholstering of all passenger and sleeping cars, including the cushions of seats and mattrasses of sleepers, shall, at least twice a week, and in the open air, as far as possible, be thoroughly whipped and brushed, and the blankets and curtains of sleepers shall be whipped and sunned thrice weekly.

"In case of infection of a car or sleeper, the bedding, mattrasses and all the upholstering shall be thoroughly disinfected before permitted to be again used."

Dr. Cobb, of Pensacola, cited cases that had been introduced by railroads.

Dr. Rauch said there was no doubt that cars could be sent from New Orleans to Chicago without danger, but doubted about points further South.

After a short discussion as to whether fever could be conveyed to a town save by direct communication with an infected town, the following important resolution was presented by Mr. Cobb, Mayor of Pensacola:

Whereas, The National Board of Health has been constituted for the purpose of investigating subjects relating to the public health, and particularly in epidemic diseases as to the best course to be pursued, which must be, in some degree, a matter of experiment in ports north of Cape Hatteras;

*Resolved*, That we recommend the petition of the delegates from the second oldest city in the Union be granted and that the National Board of Health take immediate charge and control of maratime at the port of Pensacola, in the event of the failure of Congress to provide means, and also to empower the National Board to take charge of quarantine when called upon by the municipalities.

We respectfully ask that the Navy Department be empowered to take charge of quarantine at that port rather than the maratime hospital service of the United States.

S. C. Cobb,
Mayor of Pensacola.
Geo. H. O'Neal,
President of the Board of Health.

The Board adjourned until after the address of Dr. Billings before the Medical Association.

Reassembling at 12 o'clock, Dr. Wright, of Chattanooga, resumed the discussion with thoughts suggested by experience in his own city.

Dr. White, of Milwaukee, made a speech citing rigorous measures which he, as Health Officer of Milwaukee, had instituted against the introduction of diseases.

Mr. Cobb advocated the institution of some system of ventilating freight cars.

The next subject of discussion was announced. to be, "System of notification of occurrence of first cases of dangerous diseases, especially yellow fever. When is it proper to say yellow fever is epidemic in a town ?"

Deciding that this question had already been settled by State and local authorities, the Board adjourned to meet in conjunction with the Sanitary Council of Mississippi Valley, at the Kimball House, at 8 o'clock, P.M.

---

FOURTH DAY'S SESSION—THURSDAY, MAY 8.

The final session in this city of the National Board of Health was held at the Kimball this morning, President Cabell in the Chair.

Dr. Rauch, Secretary of the Sanitary Council of the Mississippi valley, requested before the regular routine of business was entered upon, to be permitted to report to the Board a series of propositions which had been adopted by the Sanitary Council on the preceding evening. The propositions were read and left with the National Board of health for future consideration. A plan—the arrangement of which had sometime ago been referred to a committee—was then adopted securing co-operation between the National Board of Health, the Sanitary Committee of the Mississippi valley and the American Public Health Association in collating knowledge of the actual sanitary condition of every city in the United States, particularly in the Southwestern States along the Mississippi river. It is proposed to ascertain as far as possible everything that will tend to affect the sanitary condition, whether for good or evil of the various cities—the systems of sewerage, the condition of vaults and the disposition that is made of refuse matter, and from these data to compile the best sanitary regulations and correct existing abuse.

After adopting these measures the National Board of Health adjourned to meet in convention at Nashville on the 8th of November. From this it is not to be understood that the National Board of Health has literally adjourned until that date; they will, on the other hand, hold sessions during the entire summer.

From the discussions will be ascertained, as far as possible, the best sanitary measures for the prevention as well as the eradication of epidemics; these results, when obtained, will be furnished to local and State sanitary bodies. Then on the 8th of November the Board will meet again in Nashville, there to hold a conference similar to the one just held in Atlanta, with leading sanitarians invited from every quarter of the Union. Should the Board obtain an appropriation of six hundred and fifty thousand dollars from Congress as they desire, they would be enabled themselves to put into effect the results of their deliberations; but with their present means they can only accumulate knowledge, deduce sanitary measures from it and refer them to local and State authorities to be put into effect.

The Sanitary Council of the Mississippi valley and the American Health Association have agreed to meet in Nashville also on the 8th of November.

---

### THE AMERICAN MEDICAL EDITORS

Met May 5th, at 8 o'clock, in the Senate Chamber. It was called to order by President Wm. Brodie, of Detroit. Dr. Robert C. Word, of the Southern Medical Record, of Atlanta, the Assistant Secretary, was also present.

Dr. Brodie read a full, able and interesting paper on the history, scope, duty and destiny of American medica journals. He examined the subject in a masterly manner and so as to make it interesting to all who heard it. We regret that want of space will not permit a full analysis of this able paper, so full of the spirit of true progress in medical journalism. The following is a list of the members present:

Dr. L. Connor—The Detroit Lancet.

Dr. Duncan Eve, Dr. D. J. Roberts—The Southern Practitioner.

L. Frank Woodbury—Boston Medical and Surgical Journal.

W. B. Jones, M.D.—Southern Journal of Medical and Physical Science, Nashville.

Dr. E. S. Dunster—Michigan Medical News.

Dr. T. S. Powell—Southern Medical Record.

Dr. Theophilus Parvin—American Practitioner.

Dr. W. T. Goldsmith—Southern Medical Record.

Dr. A. N. Bell—The Sanitarian.

Dr. J. G. Westmoreland—Atlanta Medical and Surgical Journal.

Dr. Wm. Brodie—New Preparations.

Dr. Robert C. Word—Southern Medical Record.

Dr. Dudley S. Reynolds—The Medical Herald, of Louisville.

Dr. J. V. Shoemaker—Medical Bulletin.

Dr. E. S. Dunster moved that the resolutions relative to the advertisement of patent medicines, which were suggested by the President in his address, be adopted by the Convention. The resolutions were to be presented to the American Medical Association, and they declare the advertisement of such patent medicines in medical journals a violation of certain sections of the code of ethics which governs the members of the Convention.

Dr. Dunster's motion to adopt the resolution was agreed to.

Dr. N. S. Davis, of Chicago, said he not only deemed such compound medicines out of the pale of professional use, but also very dangerous to use.

Dr. Leartus Connor spoke of the too free use of these patent medicines in the professions, and regretted the discriminations made in copyrighted preparations. He favored the resolutions and their transmission to the American Medical Association.

Dr. Parvin spoke highly of the President's address. He paid a compliment to the country doctors of whom the President had spoken, and said these earnest men were making the literature which would be hunted up one hundred years hence. He asked that some action be taken on the death of two distinguished members of the Convention—Dr. Hayes and Dr. Waddell. He spoke of an intimate personal acquaintance with Dr. Hayes, and paid a tribute to his faithfulness and devotion. He moved the appointment of a committee on memorials of these two deceased editors. The motion was carried and the Chair appointed Drs. Parvin, Davis and Bell.

A committee appointed to select officers for the ensuing year consisted of Drs. Dunster and Reynolds. While the committee were consulting, Dr. Davis spoke of the objects of the organization. The object designed was the promotion of the best interests of medical journalism by cooperation. He said there appeared to be in medical journals little editorial force expended on the specific duties of the profession, and the methods in which it could be made more useful than it is at present. An influence started through the medical press, on many important subjects, would penetrate to almost every household in the land.

Dr. W. T. Goldsmith said he thought the Association might be enlivened by an amendment to the Constitution admitting all authors as members.

Dr. Stephen Smith, of New York, was elected a member of the Association.

Dr. Thomas S. Powell, editor of the Southern Medical Record, spoke of the high conception he had of the ethics of the profession. He had endeavored to make his journal conform to this rule and to take a broad view on all subjects. He advocated the adoption of a broad and liberal policy by all the medical editors in the country.

Dr. Shoemaker, of Philadelphia, said he did not think that the object of the Association was ever properly understood, and on that account there was a failure on the part of some editors to take a proper interest in it.

Dr. Parvin urged a new interest in the Association. The Association was organized ten years ago at New Orleans, and he thought it had done much good. Such friendly conferences as the present did good and kept up a more hearty sympathy among the editors.

Some discussion took place as to the exclusive position of certain leading journals, such as the Boston and Chicago journals. They had cut off many of their exchanges and would not give their lordly recognition to any journal that charged less than $2 per year. This tendency was very severely criticised.

Dr. Jones, of Nashville, spoke freely on this subject, and hoped for a more general system of exchange.

Dr. Frank Woodbury, of the Boston Journal, made a few remarks, in which he said he thought an injustice had been done the journal he represented. Its editorials had been widely quoted in the secular press. He defended the motives of his journal, and denied that it had adopted any exclusive system.

The Committee on the nomination of officers reported the following names as appropriate for the offices. It will be seen that they have paid Atlanta a very high compliment in the choice of a President:

For President—Dr. Thomas S. Powell, of the Southern Medical Record.

Vice-president—Dr. Frank Woodbury, of the Boston Medical Journal.

The Secretary is a permanent officer. Dr. Frank Davis, of Chicago, fills this office.

The Secretary cast the ballot, and the President announced the result.

Dr. Dudley S. Reynold said he would urge the interest of the Association in his journal, the Medical Herald.

Dr. Jones, of Nashville, moved a resolution that at the next meeting, every member report what journals had refused to exchange with them.

The resolution was adopted.

Dr. Dowell, of Galveston, favored the membership of authors in the Association.

Dr. Goldsmith offered an amendment, changing the name of the Association so as to include authors as well as editors. Under the rule, this was laid on the table for a year.

Dr. Davis moved that a Committee of three be appointed to consider how the Association could be made more effective, and also to choose certain subjects for consideration at the next meeting. The Chair appointed Drs. Davis, Parvin and Reynolds.

Dr. Powell returned, in a graceful speech, his thanks for the honor conferred on him.

A resolution of thanks to President Brodie for his address.

The Association then adjourned, after receiving and accepting an invitation to a dining to be given them at Dr. Powell's residence, on Pryor street, at 2½ o'clock, Thursday.

